# Genetic characterisation of molecular targets in carcinoma of unknown primary

**DOI:** 10.1186/s12967-018-1564-x

**Published:** 2018-07-04

**Authors:** B. Clynick, B. Dessauvagie, G. Sterrett, N. T. Harvey, R. J. N. Allcock, C. Saunders, W. Erber, K. Meehan

**Affiliations:** 1 School of Biomedical Sciences (M504), The University of Western Australia, 35 Stirling Hwy, Crawley, WA 6009 Australia; 2 PathWest Laboratory Medicine, Fiona Stanley Hospital, 11 Robin Warren Dive, Murdoch, WA 6150 Australia; 3 PathWest Laboratory Medicine, Sir Charles Gairdner Hospital, J Block, Hospital Ave, Nedlands, WA 6009 Australia; 4 Royal Perth Hospital, 197 Wellington Street, Perth, WA 6000 Australia; 5 Fiona Stanley Hospital, 11 Robin Warren Dive, Murdoch, WA 6150 Australia

**Keywords:** Carcinoma of unknown primary, CUP, Next-generation sequencing, Mutation profiling, Druggable targets, Targeted therapy

## Abstract

**Background:**

Carcinoma of unknown primary (CUP) is a metastatic epithelial malignancy in the absence of an identifiable primary tumour. Prognosis for patients with CUP is poor because treatment options are generally limited to broad spectrum chemotherapy. A shift towards personalised cancer management based on mutation profiling offers the possibility of new treatment paradigms. This study has explored whether actionable, oncogenic driver mutations are present in CUP that have potential to better inform treatment decisions.

**Methods:**

Carcinoma of unknown primary cases (n = 21) were selected and DNA was isolated from formalin-fixed paraffin embedded sections prior to amplification and sequencing. Two distinct yet complementary targeted gene panels were used to assess variants in up to 76 known cancer-related genes for the identification of biologically relevant and actionable mutations.

**Results:**

Variants were detected in 17/21 cases (81%) of which 11 (52%) were potentially actionable with drugs currently approved for use in known primary cancer types or undergoing clinical trials. The most common variants detected were in *TP53* (47%), *KRAS* (12%), *MET* (12%) and *MYC* (12%). Differences at the molecular level were seen between common CUP histological subtypes. CUP adenocarcinomas and poorly differentiated carcinomas harboured the highest frequency of variants in genes involved in signal transduction pathways (e.g. *MET*, *EGFR*, *HRAS*, *KRAS*, and *BRAF*). In contrast, squamous cell carcinoma exhibited a higher frequency of variants in cell cycle control and DNA repair genes (e.g. *TP53*, *CDKN2A* and *MLH1*).

**Conclusion:**

Taken together, mutations in biologically relevant genes were detected in the vast majority of CUP tumours, of which half provided a potentially novel treatment option not generally considered in CUP.

**Electronic supplementary material:**

The online version of this article (10.1186/s12967-018-1564-x) contains supplementary material, which is available to authorized users.

## Background

Carcinoma of unknown primary (CUP) is classified as any type of metastatic epithelial tumour where, following extensive clinical history, physical examination, radiological studies and histopathological (including immunohistochemical) investigations, no primary site can be identified [1]. CUP is the eighth most common cancer diagnosed and is the fourth most common cause of cancer-related death in both sexes worldwide [2–4]. The overall age-standardised incidence of CUP ranges between 4 and 9 cases per 100,000 people annually worldwide [3–5]. CUP is an aggressive, highly heterogeneous disease with a variable biology. There is no standard treatment and broad-spectrum chemotherapy (e.g. paclitaxel, carboplatin) is generally used [6, 7]. Clinical trials are difficult to perform because of the heterogeneity in tumour types within CUP. As a result, CUP has a poor prognosis with a median survival of less than 12 months and 5-year survival only 14% [8]. There is an urgent need to improve treatment and prolong survival for patients with CUP. The new era of personalised medicine through the use of next-generation sequencing (NGS) technologies offers such an opportunity based on the identification of targeted therapies [9].

Next-generation sequencing technology allows whole-genome sequencing, whole-exome sequencing, or mutation analysis with specific (“targeted”) panels of genes. Many genomic studies are limited by the availability of poor quality formalin-fixed paraffin-embedded (FFPE) tissue which make up the main source of sample preparation and storage in routine diagnostics [10, 11]. As such, for NGS to be useful in the clinical setting, small amounts of FFPE tissue from variable sources needs to be successfully evaluated. With advancements in knowledge about important targetable mutations across various cancers, targeted sequencing approaches allow selective screening of known druggable targets using relatively small amounts of DNA input. Focusing on specific regions of interest through targeted sequencing leads to a greater depth of coverage, increasing the confidence of identifying low-level variants in cancer samples [10, 12].

Recent studies have demonstrated that precision medicine may play a crucial role in optimising treatment for patients with malignant disease [13–16]. Furthermore studies have shown improved overall survival in patients with advanced cancers who have received genotype-matched targeted therapies [15, 17–19]. This approach, if applied to CUP, may lead to personalised approaches to treatment by targeting tumour-specific somatic variants. This could become highly relevant in CUP, as genomic studies have revealed that these metastatic tumours commonly have a complex mutational landscape [3, 20]. In the present study, we assessed the genomic profile of CUP to provide insight into the genetic makeup of these tumours, and determined whether there were potentially actionable targets by performing targeted NGS.

## Methods

### Patients and samples

Formalin-fixed paraffin-embedded specimens from 21 cases of CUP were included in the study. Cases were selected based on the archival histopathological report and subsequent review by experienced pathologists. Cases were diagnosed as CUP following review of clinicopathological details and a complete histopathological work-up [including immunohistochemistry (IHC)] revealed a metastatic lesion without a specific site of origin. The clinicopathological details of cases are outlined in Table 1. Ethical approval for this study was obtained from the Sir Charles Gardiner Hospital Human Research Ethics Committee (SCGH HREC Number 2014-025) and the Department of Health WA Human Research Ethics Committee (DOH HREC Number 2015-40).

**Table 1:** Summary of the clinicopathological characteristics

Characteristic	Number of cases
All cases	21
Age (years)	
Median (range)	71 (36–91)
Female/male	14/7
Histologic characteristics	
Squamous cell carcinoma	8
Poorly differentiated carcinoma	6
Adenocarcinoma	5
Neuroendocrine carcinoma	2
Anatomical location	
Bladder	1
Bone	2
Brain	1
Liver	1
Lymph node^a^	8
Maxillary sinus	1
Omentum	1
Parotid	2
Pleura	1
Skin	1
Submandibular gland	1
Thyroid	1

^a^Axillary, cervical, inguinal, intraparotid, mesenteric, neck, retroperitoneal

### Tumour cell isolation from FFPE specimens

The tumour content of the FFPE samples was assessed on haematoxylin and eosin (H&E) stained slides. Each case was processed in three different ways depending on the percentage of tumour content. For cases with > 70% tumour content (n = 11), 2 × 10 µM sections were collected in microcentrifuge tubes (Axygen, Australia) for DNA extraction. For cases with < 70% tumour content (n = 21), matched H&E slides were used to guide either macro or micro-dissection of tumour rich regions. Specifically, for cases with 50–70% tumour content (n = 6), macro-dissection was performed to obtain > 90% tumour rich material for DNA extraction. In brief, sections were mounted onto double positive charged slides (Hurst Scientific, Australia) and air-dried for 1 h. A sterile scalpel blade was used to scrape the tumour-rich tissue off the unstained slides into LoBind Eppendorf tubes (Eppendorf, Australia) for DNA extraction. Micro-dissection was performed on cases with < 50% tumour content (n = 4) using 4 × 8 µM sections. Briefly, each slide was de-paraffinised, stained and dehydrated through a series of xylene/ethanol washes. Tumour rich areas were micro-dissected using an ArcturusXT laser capture micro-dissection (LCM) instrument (ThermoFisher Scientific, USA) with CapSure Macro LCM caps (ThermoFisher Scientific, USA). Each section was selectively captured by focal melting of the caps polymer membrane with a small to medium sized infrared (IR) pulse (range between 45 and 82 mW) and an ultra-violet (UV) laser beam (range between 5 and 35 mW/ms). The power and duration of the laser were adjusted each time a new section of tissue was selected.

### DNA extraction

For all three tissue processing methods, genomic DNA was isolated using a GeneRead DNA FFPE kit (Qiagen, Germany) according to manufacturer’s instructions, with minor modifications. In brief, once the tissue from each specimen was de-paraffinised, samples were incubated at 56 °C for 1 h with proteinase K, then again at 90 °C for 1 h to partially reverse formaldehyde modification of nucleic acids. Following tissue digestion, the samples were treated with Uracil-DNA-Glycosylase (UNG; Qiagen, Germany) for the specific removal of artificially induced uracils introduced by fixation and embedding. Silica-gel membrane spin columns were then used to bind the DNA, facilitating the removal of any contaminants. Purified DNA was then eluted from the spin column using nuclease-free water to minimise salt carry-over. The DNA template was quantitatively assessed using a Qubit Fluorometer (ThermoFisher Scientific, USA) and Qubit dsDNA HS Assay Kit (Life Technologies) according to manufacturer’s instructions.

### Library preparation

Two different targeted panels were used to generate sequencing libraries from 10 ng of DNA. The Oncomine Focus Assay (OFA) and Cancer Hotspot v2 (CHPv2) panel (Thermofisher Scientific, USA) are specifically optimised for detection of up to 50 genes commonly implicated in human cancers and relevant to targeted treatment of solid tumours (Additional file 1: Table S1). Library preparation for each sample differed slightly between the two panels. Several OFA libraries and one CHPv2 assay library were prepared using Ion PGM Select and Ion AmpliSeq reagents (ThermoFisher Scientific, USA) respectively, according to the manufacturer’s instructions. For both panels, unique barcode adapters 1–32 (Ion PGM Select Adaptors; ThermoFisher Scientific, USA) were ligated to the amplicons and subsequently purified to ensure each individual sample had a unique ID. The final amplicon libraries were then amplified, purified and equalised to ~ 100 pM using AMPure beads (Ion PGM Select Library Equaliser; ThermoFisher Scientific, USA).

### Emulsion PCR and semiconductor sequencing

Uniquely barcoded library samples were pooled for sequencing on either an Ion 318 chip (for the OFA) or an Ion PI v3 chip (for the CHPv2 panel). Each pool was clonally amplified onto Ion Sphere Particles (TMPL ISP; Ion OneTouch Select Template Reagents, ThermoFisher Scientific, USA) by emulsion PCR. For the OFA this was carried out using a One Touch 2 System (ThermoFisher Scientific, USA), and each pool was manually loaded onto an Ion 318 Select chip. In contrast, for the CHPv2 assay, the Ion Chef System (ThermoFisher Scientific, USA) was used for fully automated template preparation and Ion PI v3 chip loading. Single-end sequence analysis was carried out either on the Ion PGM (OFA) or Ion Proton Sequencer (Cancer Hotpsot v2) (ThermoFisher Scientific) for 200-base-read-sequencing.

### Coverage and data analysis

Raw data from both panels was collected, processed and trimmed using the Ion Torrent platform-specific software. Removal of polyclonal and low-quality reads, as well as 3′ quality trimming of reads was performed using TorrentSuite v4.6 (ThermoFisher Scientific, USA). Reads were aligned to the reference genome (human genome hg19) and Ion Reporter v5.0 software package (ThermoFisher Scientific, USA) was used to detect and annotate variants for both panels. Specifically, the ‘Oncomine Focus Panel v1—DNA—Single Sample’ automatic workflow in Ion Reporter was used to identify and annotate the copy number variants from the OFA. This workflow has preconfigured parameter settings for copy number calling, including a 5% confidence interval and CNV ploidy ≥  gain of 2 over normal. Alternatively, the ‘CHPv2—Annotate variants single sample’ automatic workflow was used to identify and annotate variants from the CHPv2. Ion Reporter was also used to identify a subset of variants previously reported in publicly available databases (namely, the 1000 Genomes Project). The resulting annotated variant data detected with the OFA were further analysed using the Ion Torrent Oncomine Knowledgebase Reporter v2.0.3 (ThermoFisher Scientific, USA), providing details of the clinically relevant targeted therapies currently on the market or undergoing clinical trials for the associated Oncomine variants detected.

## Results

The cohort comprised 67% (14/21) female and 33% (7/21) male patients with a median age at diagnosis of 71 (range 36–91). Tumours were classified into four histological subgroups: squamous cell carcinoma (SCC; 38%), poorly differentiated (PD) carcinoma (29%), adenocarcinoma (24%), and neuroendocrine carcinoma (9%). The most common anatomical site where CUP presented was in lymph nodes (38%) (Table 1).

DNA was extracted from tissue sections of 11 cases, macro-dissected tissue from 6 cases and LCM tissue from 4 cases (Table 2). The concentration of DNA collected ranged between 0.5 and 176 ng/µL which varied depending on tumour cell isolation type. The average concentration of DNA extracted from tissue sections was 45 ng/µL (range 3–176 ng/µL); from macro-dissected tumour tissue it was 84 ng/µL (range 36–157 ng/µL); and from LCM cases it was 3 ng/µL (range 0.5–10 ng/µL). Although DNA concentration varied by sampling method, there was a sufficient amount of DNA extracted for sequencing with both panels, with the exception of LCM cases which were re-captured and re-extracted for the second sequencing panel.

**Table 2:** Clinicopathological and genetic characteristics of the 17 cases with biologically relevant or actionable variants

Sample ID	Anatomical location	Morphology	Gender	Age	% of tumour cellularity	Sampling method	Gene	Amino acid change	Copy number detected	Allele ratio	Detected by both Panels	Actionable target
1	Lymph node (cervical)	SCC	F	62	80	TS	*TP53*	G245D		0.79		
							*CCND1*		12.31			Yes
							*FGFR1*		8.31			Yes
							*MYC*		10			Yes
2	Lymph node (inguinal)	SCC	M	67	90	TS	*TP53*	I195N		0.13		
3	Lymph node (inguinal)	SCC	F	51	100	Macro	*CDKN2A*	D74N		0.35		
							*PIK3CA*	E545K		0.17	Yes	Yes
4	Lymph node (mesenteric)	PD Carcinoma	F	81	100	LCM	*BRAF*	V600E		0.22	Yes	Yes
5	Maxillary sinus	SCC	F	79	90	TS	*TP53*	R248W		0.23		
							*TP53*	P177L		0.29		
							*ERBB2*	S310F		0.28		Yes
6	Parotid	SCC	M	54	100	Macro	*TP53*	E286K		0.08		
7	Skin	Neuroendocrine Ca	F	81	95	TS	*MET*	R988C		0.4		Yes
8	Submandibular gland	SCC	M	81	95	TS	*MYC*		6.97			Yes
9	Bone	PD Carcinoma	F	80	50	TS	*MET*	T1010I		0.49	Yes	Yes
10	Bone	Adenocarcinoma	M	71	100	LCM	*KRAS*	G12D		0.67	Yes	Yes
11	Lymph node (axillary)	Adenocarcinoma	F	82	80	TS	*TP53*	Q165Ter		0.51		
12	Omentum	Adenocarcinoma	F	68	100	LCM	*TP53*	R175H		0.75		
13	Parotid	SCC	M	79	100	Macro	*TP53*	S241F		0.28		
							*TP53*	P152L		0.22		
14	Parotid	PD Carcinoma	F	76	100	Macro	*HRAS*	G13N		0.51	Yes	Yes
							*HRAS*	G13S		0.51	Yes	Yes
15	Thyroid	SCC	F	80	100	Macro	*MLH1*	V384D		0.5		
							*BRAF*	V600E		0.12	Yes	Yes
16	Brain	PD Carcinoma	M	74	80	TS	*KRAS*	G12A		0.61	Yes	Yes
17	Liver	PD Carcinoma	F	66	80	TS	*TP53*	R175H		0.57		

Characteristics of the CUP cases includes the sample ID, anatomical location where the CUP originated from, the histopathological morphology of the specimen, the gene the variant was detected in, the protein change, total number of gene copies detected, the allele ratio of single nucleotide variants (SNVs), and whether the variant identified was actionable

*SCC* squamous cell carcinoma, *F* female, *M* male, *PD* poorly differentiated, *Ca* carcinoma, *TS* tissue sections, *macro* macrodissection, *LCM* laser capture microdissection

With the OFA, all samples were sequenced to an average mean depth of 1396 reads per nucleotide position within the ~ 27 kb target region, whereas with the CHPv2 panel, the samples were sequenced to an average mean depth of 9105. The distribution of reads across the 269 amplicons was consistent among samples from both panels, with an average uniformity coverage of 98.25% and 98.81% for the OFA and CHPv2 panels respectively. Approximately 94% of the sequence reads from both panels were mapped to the targeted gene regions (aligned to human genome reference 19), demonstrating the high specificity of the amplicon-based amplification method. Successful sequencing of the samples was measured by using a minimum of 250,000 reads with a quality score of AQ20, a sequencing coverage of 1000×, as well as a variant frequency of at least 5% in a background of wildtype alleles.

**Fig. 1:**
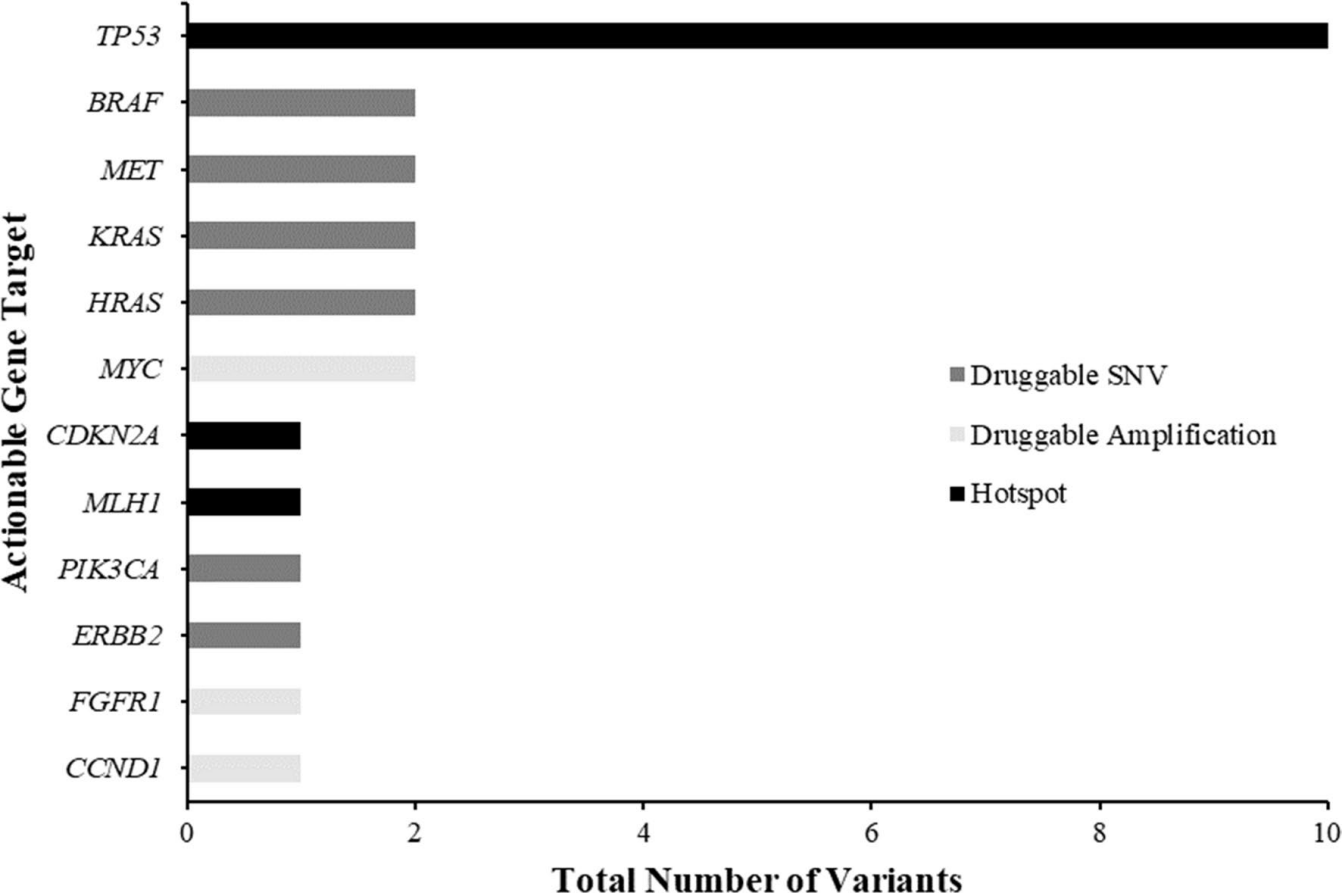
Frequency of clinically relevant gene targets. Total number of druggable single nucleotide variants (SNVs) (dark grey), druggable amplifications (light grey) and hotspot mutations (black) identified across the CUP cases (total number of variants n = 26). *BRAF*, *MET*, *KRAS* and *HRAS* were the most common druggable SNVs; *MYC* was the most common druggable copy number variant (CNV) detected; and *TP53* was the most common hotspot gene detected

From the combined results of both panels, a total of 608 variants were detected in 41 genes (10 genes for which no variants were identified in), with an average of 14 variants detected per case (range 0–26 variants). Stringent variant detection criteria were used to identify likely somatic variants. First, we filtered out variants with a minor allele frequency (MAF) > 5% (according to the 1000 Genomes Project) and synonymous exonic mutations. After frequency filtering, a total of 26 variants remained, of which 14 were known gain-of-function variants; 10 actionable single nucleotide variants, and 4 actionable copy number variants (Fig. 1); the remaining 12 variants were known cancer related hotspot variants. The allele ratios at which the hotspot variants occurred at ranged from 0.08 to 0.79. Six samples had reportable variants within regions covered by both panels, and these variants were detected at similar allele frequencies, emphasising the validity of the variants detected (Table 2).

Variants were identified in cases with DNA isolated by all three methods. Specifically, variants were identified in 9 cases with DNA extracted from tissue section, in 5 cases from macro-dissected tissue and in 3 cases with DNA extracted from LCM tissue (Table 2). There was no correlation between the number of variants and allele frequencies detected and the different percentages of tumour cellularity (50–100%) or the sampling methods used (tissue sections versus macro-dissection versus LCM) (Table 2).

**Fig. 2:**
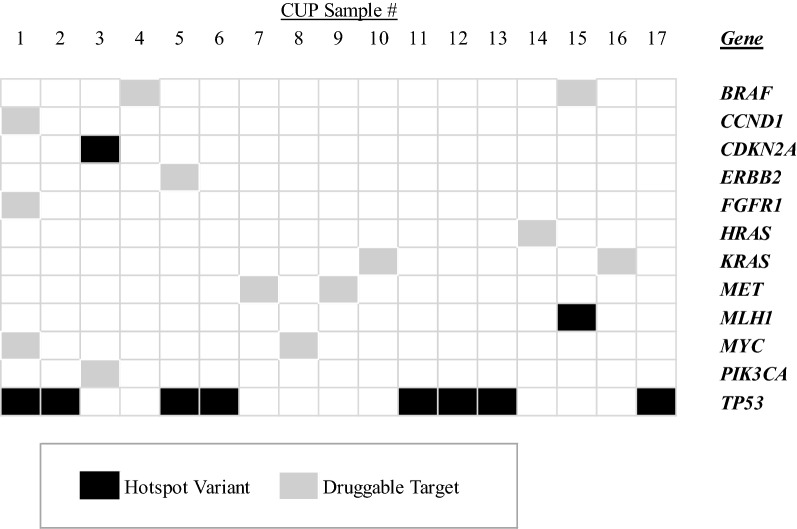
Integrated gene map of the variant data from the Oncomine Focus Assay panels (OFA) and Cancer Hotspot v2 (CHPv2) panel (ThermoFisher Scientific, USA) identified in the CUP cohort. This gene map shows the pattern of case-specific, concurrent and mutually exclusive mutations identified in the CUP cases. Each column represents an individual case and each row denotes a specific gene assigned to one of two functional categories (black—hotspot variant; grey—druggable target). The list of genes includes only those found to be aberrantly expressed within the cohort, and represents a subset of the total number of genes interrogated by both panels. No variants were identified in four cases and were not included in the figure

Following the filtering process, biologically relevant and therapeutically actionable variants were identified in 81% (17/21) of the cohort. The mean number of variants was 1 per case (range 0–4) (Table 2 and Fig. 2). Hotspot mutations were identified in 59% (10/17) of cases. These were in genes associated with various cell signalling, cell cycle control and DNA repair pathways. The gene with the most common hotspot mutations was *TP53* identified in 47% (8/17) of cases, and the most common copy number variation (CNV) was *MYC* amplification identified in 12% (2/17) of cases. Correlation with the tumour pathology showed that the CUP cases with morphology of adenocarcinoma and PD carcinoma had the largest number of cell signalling pathway variants (*EGFR*, *MET*, *JAK3*, *KRAS*, *HRAS*, *BRAF*, *PIK3CA*, *PTPN11* and *APC*). In contrast, SCC tumours showed a higher number of variants in cell cycle regulation genes (*TP53* and *CDKN2A*). There were no associations with other clinicopathological parameters (i.e. age, gender or anatomical site of presentation).

Potentially actionable targets were detected in 52% (11/21) of cases. The most common variants were in the *KRAS*, *HRAS* and *BRAF* genes. Although the mean number of actionable variants was 1 per case (range 1–3), one sample presented with 3 possible druggable targets (*CCND1*, *FGFR1* and *MYC*) (Table 2). When the number of variants were normalised by site (i.e. $\frac{{ {\text{number of cases with variants presenting in a site}}}}{\text{total number of CUP cases }} \times {\text{number of variants detected in that site}}$), the highest number of variants were detected in lymph nodes (all lymph nodes) (0.7 variants), followed by brain (0.2 variants). When the number of variants were normalised by histological morphology, the subtype associated with the greatest number of variants was SCC (1.8 variants), followed by PD carcinoma (0.8 variants). When the number of variants were normalised by gender, the highest number of variants were detected in female CUP patients (3.8 variants) compared with male CUP patients (0.4 variants).

## Discussion

A recent shift in treatment focus towards personalising cancer management has encouraged mutational profiling [21–24]. This is the first study to utilise two complementary gene panels to identify variants in CUP that are aligned to known oncogenic driver mutations and approved therapies with published evidence of targeted on-the-market drugs or therapeutics currently in clinical trial. We have identified relevant variants in 81% (n = 17) of CUP cases, of which over half (65%) were to potentially actionable targets. This is a highly significant result as the identification of variants for which there is a known therapeutic agent available may offer a potential new and “personalised” treatment approach for patients with CUP. This is important considering the limited therapeutic benefit current CUP patients receive with generic chemotherapy. Support for this concept is offered by several small studies of patients with CUP that have reported durable treatment responses with the use of mutation matched (e.g. *EGFR*, *KIT*, *MET* and *BRAF*) targeted therapies [18, 19, 25–28]. Currently approved existing therapeutic agents are available for 2 of the mutations detected (*BRAF* V600E, *ERBB2* S310F), whilst therapeutic agents for the other gain-of-function variants detected (i.e. *CCND1*, *FGFR1*, *MYC*, *PIK3CA*, *MET*, *KRAS* and *HRAS*) are currently being investigated in active, ongoing clinical trials. A large proportion of the variants detected in this study are known to be associated with various signal transduction pathways, apoptotic regulation and cell cycle progression. These results are promising as the majority of available targeted drugs target act through one of these pathways, which are commonly altered in many cancers [29–31].

The most commonly mutated gene identified in this study was *TP53* (47%, 8/17) with 9 different non-synonymous coding region variants. This is unsurprising, because *TP53* mutations have been described to contribute to metastatic progression in multiple cancer types, supporting the high percentage of *TP53* variants reported in CUP [32]. Other common variants detected in this cohort were observed in genes involved in the activation and regulation of key signal transduction pathways (i.e. *BRAF*, *HRAS* and *KRAS*). This is the first study to report *HRAS* variants and codon 12 *KRAS* variants (G12A) in CUP [22, 23]. Although activating mutations in codons 12 and 13 of *KRAS* are the most commonly occurring isoforms in human cancers, variants were limited to codon 12 in the present cohort [33, 34]. *KRAS* codon 12 mutations confer a more aggressive tumour phenotype with stronger transforming abilities compared with codon 13 mutations [33, 35–37]. The detection of codon 12 mutations in this cohort is consistent with the highly aggressive nature of CUP tumours. Furthermore, characterising the mutational status of *KRAS* has become clinically relevant in some malignancies, because the presence of a *KRAS* mutation is known to confer poor response to some tyrosine kinase inhibitors (e.g. EGFR inhibitors) [38, 39]. Although there is currently no therapeutic agent to target and inhibit mutant *KRAS* activity, a recent case study reported a partial response in a CUP patient treated with a MEK inhibitor (trametinib) following the detection of a *KRAS* G12D mutation [28, 40]. This encourages the detection of *KRAS* as a possible druggable target in CUP.

Activating *BRAF* V600E mutations were identified, in keeping with other reports [6, 21, 22, 24]. This offers the prospect of treatment with BRAF inhibitors (e.g. vemurafenib and dabrafenib) for CUP with *BRAF* V600E mutations. This has been exemplified in a case of CUP with a *BRAF* V600E inguinal nodal mass mutation that showed successful treatment (complete clinical response) with BRAF targeted therapy (vemurafenib) coupled with immunotherapy (ipilimumab) [25]. Mutations in *MET* and *ERBB2* were detected in 3 cases, providing the possibility of targeting these receptor tyrosine kinases (RTK). Targeted MET therapy (crizotinib) has been used with success in CUP patients in combination with HER2 targeted therapy (trastuzumab). The current success of HER2 and MET targeted therapies in advanced and/or metastatic malignancies, and the recent success of trastuzumab and crizotinib demonstrating a positive response in a HER2 and *MET*-mutant CUP tumour, provides evidence for future evaluation of these genes as druggable targets in patients with CUP.

Our results support those of other CUP studies which have demonstrated the value of sequencing techniques for the identification of actionable targets [6, 14, 21, 23]. These studies similarly identified actionable variants in 75% (n = 16), 85% (n = 200), 55% (n = 87) and 30% (n = 150) of CUP cases, including activating variants in core mitogenic and cell growth pathways. Comparable to our study, the most common clinically relevant alterations detected by these studies included *ERBB2*, *EGFR*, *KRAS*, *PIK3CA* and *BRAF*. Previous studies detected additional actionable variants in other genes involved in cell proliferation, cell cycle progression and apoptotic regulation (*AKT1*, *FGFR3*, *JAK2*, *BRCA1*, *PTEN*, *RICTOR*, *NF1*, *CDKN2A*, *CTNNB1* and *MCL1*). Variants in these genes may have not been detected in the present study due to the use of specific gene panels, one of which enables a more translatable definition of actionable ability, which is not taken into consideration by prior CUP sequencing studies [6, 21, 23, 24].

This is the first study to have successfully compared different tissue processing techniques (i.e. tissue sections versus macro-dissection versus LCM) for the enrichment of tumour cells from CUP tissue with the subsequent detection of actionable targets by targeted NGS. Variants were successfully identified in cases with DNA isolated by all three methods, highlighting the use of low input DNA for accurate targeted sequencing. Previous studies have used hybridisation capture techniques that are optimal for samples possessing a high tumour burden, requiring larger concentrations of input DNA (minimum of 50 ng). The use of an amplicon-based approach (as used in this study) tolerates even lower concentrations of input DNA and is advantageous because it is amenable to sequencing low levels of enriched tumour cell populations (isolated by LCM). This is highly relevant in the context of CUP, because a large proportion of CUP cases have limited tissue available as they are generally core biopsies and fine needle aspirate FFPE cell blocks. Furthermore, amplicon targeted sequencing has a quicker turnaround processing time, leading to less expensive sequencing costs compared with hybridization capture methods, proving diagnostically feasible for routine molecular pathology laboratories [10, 12].

**Fig. 3:**
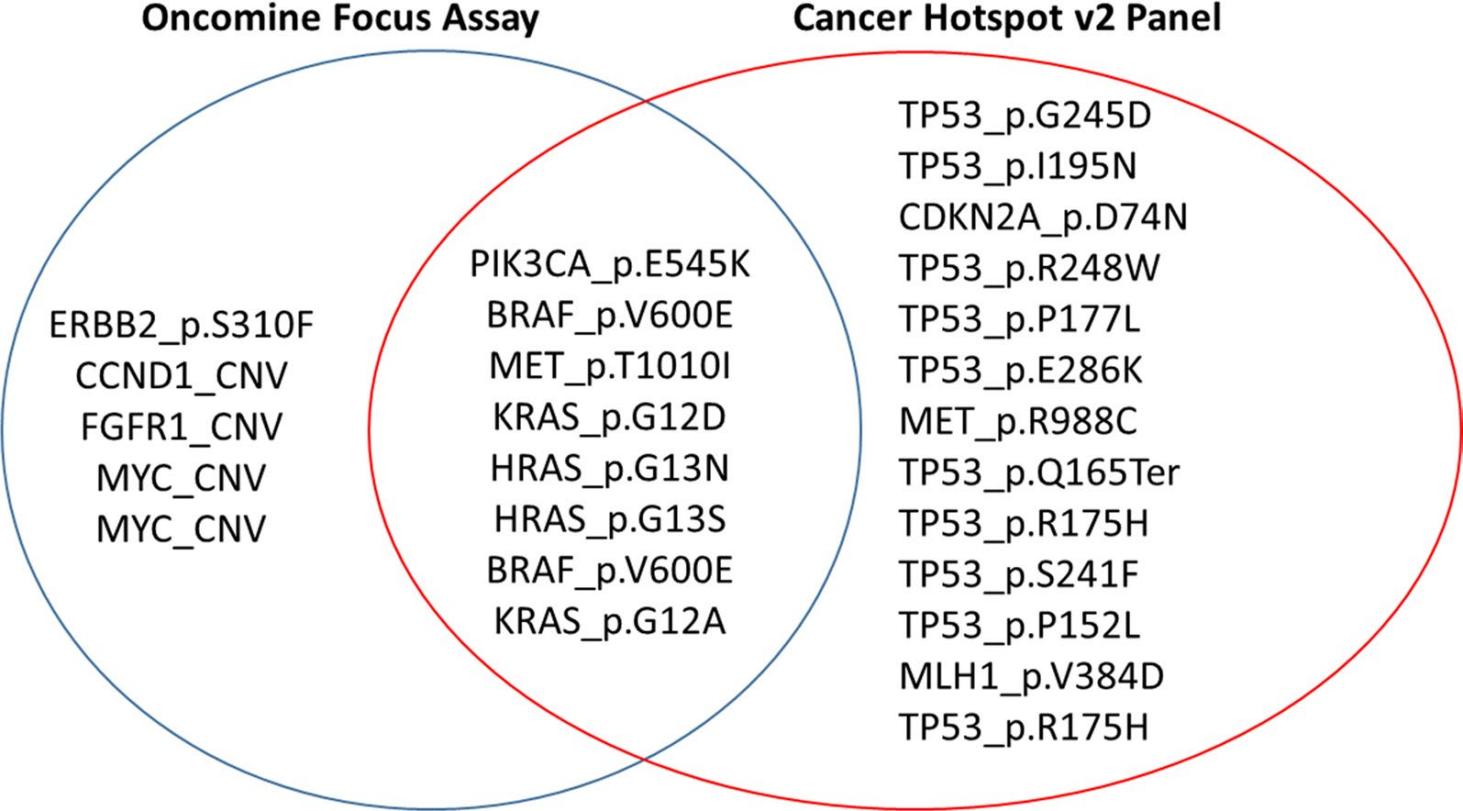
Venn diagram representing the variants exclusively detected by each panel, and the overlap of variants detected by both panels. Five variants were only detected by the Oncomine Focus Assay (OFA); 13 variants were only detected by the Cancer Hotspot v2 panel (CHPv2); 8 variants were commonly detected by both panels

The use of two different but complementary NGS panels provided an internal, orthogonal method for validating the sequencing results, whereby several variants covered by both panels were similarly identified (Fig. 3). We demonstrated that variants detected in our study that were included in both the CHPv2 and OFA panels were 100% concordant. This confirms that the workflow and platform utilised in this study with either panel generated results that were reproductible, accurate and precise to each sample. Seven different variants covered by both sequencing panels were detected in six different CUP specimens. Specifically, this included common hotspot variants in *PIK3CA*, *MET*, *HRAS*, *KRAS*, and *BRAF* that were detected at similar allele frequencies (Table 2). Although 8 hotspot targets were identified by both panels (Fig. 3), other important hotspot variants commonly reported in cancer were found exclusively by either panel. For example, the detection of *ERBB2* p.310F gain-of-function variant was only covered by the OFA, whilst *TP53* and *CDKN2A* hotspot variants were only covered by the CHPv2 panel. It is important to note that additional clinically relevant copy number alterations were detected by the OFA panel only. This included the detection of amplifications (8 or more copies) in *CCND1*, *FGFR1* and *MYC* across two different CUP cases.

Although mutations in driver genes were identified in the vast majority of cases (81%, 17/21), there was no single common CUP-specific molecular profile. In fact, over 80% of cases harboured exclusive variants. This highlights the genetic heterogeneity of CUP and supports the theory that these are not a discrete group of malignancies. They have different clinico-pathological characteristics which may be modulated by distinct biological mechanisms differing at a molecular level [21, 41, 42]. In support of this, and despite the small number of cases analysed, we did identify some difference in variants between pathological subtypes. Adenocarcinoma and PD carcinoma more commonly showed variants in genes involved in signal transduction pathways (e.g. *MET*, *JAK3*, *EGFR*, *HRAS*, *KRAS* and *BRAF*), whereas alterations in cell cycle control and DNA repair pathway genes (e.g. *TP53*, *CDKN2A* and *MLH1*) were more commonly seen in SCC. This is in keeping with other reports assessing histological subtypes in a range of different cancers including CUP [21, 43–45]. This not only indicates biological differences but could suggest differences in therapeutic responses. However, further studies are needed to expand and elucidate the relationship between specific mutations in CUP presenting at differing anatomical sites and the efficacy of targeted drug activity, as it is well established that, for example, not all *BRAF* mutant malignancies respond to BRAF targeted therapies [46–49].

## Conclusion

The poorly differentiated nature of CUP tumours and lack of specific antigen detection, prevents primary tissue of origin diagnoses in these patients. Without the identification of a primary origin site, treatment is restricted to generic chemotherapy with limited benefit. The detection of mutations across the majority of CUP cases included in this study highlights not only the genomic instability present in these tumours, but also offers the possibility of targeted therapies for a significant percentage of patients with CUP. The opportunity for alternative therapeutic options has the potential to improve the prognosis for CUP. Identification of actionable targets could prove useful in complementing routine diagnostic work-up and guiding therapeutic decisions for patients with CUP.

## Additional file

**Additional file 1: Table S1.** Combined gene list of the Oncomine Focus Assay (OFA) and Cancer Hotspot v2 (CHPv2) panel (ThermoFisher Scientific).

## Abbreviations

CNV: copy number variation
CHPv2: cancer hotspot v2 panel
CUP: carcinoma of unknown primary
DOH: Department of Health
FFPE: formalin-fixed paraffin embedded
H&E: haematoxylin and eosin
HREC: Human Research Ethics Committee
IHC: immunohistochemistry
IR: infrared
ISP: ion sphere particles
LCM: laser capture micro-dissection
MAF: minor allele frequency
NGS: next-generation sequencing
OFA: oncomine focus assay
PD: poorly differentiated
SCC: squamous cell carcinoma
SCGH: Sir Charles Gardiner Hospital
SNVs: single nucleotide variants
UNG: uracil-DNA-glycosylase
UV: ultra-violet
WA: Western Australian
