# Supplementary material for: Genetic characterisation of molecular targets in carcinoma of unknown primary
Source: J Transl Med. 2018 Jul 4;16:185. doi: 10.1186/s12967-018-1564-x (PMC6032776; doi:10.1186/s12967-018-1564-x)
Supplement: Supplementary file 1 — Additional file 1: Table S1. Combined gene list of the Oncomine Focus Assay (OFA) and Cancer Hotspot v2 (CHPv2) panel (ThermoFisher Scientific). [file 12967_2018_1564_MOESM1_ESM.docx]

Table S1. Combined gene list of the Oncomine Focus Assay (OFA) and Cancer Hotspot v2 (CHPv2) panel (ThermoFisher Scientific).

| ONCOMINE FOCUS ASSAY | | | | |  |  | CANCER HOTSPOT V2 PANEL | |
| --- | --- | --- | --- | --- | --- | --- | --- | --- |
| Hotspots | Copy Number Variants | | Fusion Drivers | | Overlapping Hotspots | | Hotspots | |
| *AR* | *ALK* | *KRAS* | *ABL1* | *FGFR3* | *AKT1* | *IDH1* | *ABL1* | *MLH1* |
| *CDK4* | *AR* | *MET* | *AKT3* | *MET* | *ALK* | *IDH2* | *APC* | *MPL* |
| *DDR2* | *BRAF* | *MYC* | *ALK* | *NTRK1* | *BRAF* | *JAK2* | *ATM* | *NOTCH1* |
| *ERBB3* | *CCND1* | *MYCN* | *AXL* | *NTRK2* | *CTNNB1* | *JAK3* | *CDH1* | *NPM1* |
| *ESR1* | *CDK4* | *PDGFRA* | *BRAF* | *NTRK3* | *EGFR* | *KIT* | *CDKN2A* | *PTEN* |
| *JAK1* | *CDK6* | *PIK3CA* | *ERG* | *PDGFRA* | *ERBB2* | *KRAS* | *CSF1R* | *PTPN11* |
| *MAP2K1* | *EGFR* |  | *ETV1* | *PPARG* | *ERBB4* | *MET* | *EZH2* | *RB1* |
| *MAP2K2* | *ERBB2* |  | *ETV4* | *RAF1* | *FGFR2* | *NRAS* | *FBXW7* | *SMAD4* |
| *MTOR* | *FGFR1* |  | *ETV5* | *RET* | *FGFR3* | *PDGFRA* | *FGFR1* | *SMARCB1* |
| *RAF1* | *FGFR2* |  | *EGFR* | *ROS1* | *GNA11* | *PIK3CA* | *FLT3* | *SRC* |
| *ROS1* | *FGFR3* |  | *ERBB2* |  | *GNAQ* | *RET* | *GNAS* | *STK11* |
|  | *FGFR4* |  | *FGFR1* |  | *HRAS* | *SMO* | *HNF1A* | *TP53* |
|  | *KIT* |  | *FGFR2* |  |  |  | *KDR* | *VHL* |
|  |  |  |  |  |  |  |  |  |

The OFA amplifies 35 gain-of-function genes targets, 19 CNV and 23 fusion driver genes, whereas the CHPv2 panel amplifies 50 target genes. Both panels have 24 overlapping gene targets.
